# Carotenoids from Cyanobacteria: A Biotechnological Approach for the Topical Treatment of Psoriasis

**DOI:** 10.3390/microorganisms8020302

**Published:** 2020-02-21

**Authors:** Graciliana Lopes, Duarte Clarinha, Vitor Vasconcelos

**Affiliations:** 1 CIIMAR/CIMAR, Interdisciplinary Centre of Marine and Environmental Research, Novo Edifício do Terminal de Cruzeiros do Porto de Leixões, Avenida General Norton de Matos, S/N, 4450-208 Matosinhos, Portugal; duartenunocl@gmail.com (D.C.); vmvascon@fc.up.pt (V.V.); 2 FCUP, Department of Biology, Faculty of Sciences, University of Porto, Rua do Campo Alegre, 4169-007 Porto, Portugal

**Keywords:** carotenoids, cyanobacteria, inflammation, keratinocytes, oxidative stress, psoriasis

## Abstract

In this study, five cyanobacteria strains (*Alkalinema aff. pantanalense* LEGE15481, *Cyanobium gracile* LEGE12431, *Nodosilinea* (*Leptolyngbya*) *antarctica* LEGE13457, *Cuspidothrix issatschenkoi* LEGE03282 and *Leptolyngbya*-like sp. LEGE13412) from the Blue Biotechnology and Ecotoxicology Culture Collection (LEGE CC) of CIIMAR were explored for their biotechnological potential in the treatment of psoriasis. Different extracts were characterized for their pigment profile by HPLC-PDA. The antioxidant potential of the extracts was assessed against the superoxide anion radical (O_2_^•-^). Their anti-inflammatory and antiproliferative potential was assessed in vitro using the macrophages RAW 264.7 and the human keratinocytes HaCaT as cell-line models, respectively. Terrestrial and freshwater strains presented the highest carotenoid content (33193−63926 μg/g dry extract), with all-*trans-β*-carotene, zeaxanthin, echinenone and lutein derivatives being the most abundant carotenoids. Acetone was the most effective solvent for pigment extraction. The acetone extracts presented the lowest IC_50_ values (0.29−0.38 mg dry extract/mL) regarding O_2_^•-^ scavenging, and revealed anti-inflammatory potential, with *N. antarctica* LEGE13457, *A. pantanalense* LEGE15481 and *Leptolyngbya*-like sp. LEGE13412 reducing the nitric oxide (NO) in RAW 264.7 cell culture medium in about 25% (*p* < 0.05). With the exception of *A. pantanalense* LEGE15481, all the extracts significantly reduced keratinocyte proliferation (*p* < 0.05), demonstrating a selective toxicity among the different cell lines. Overall, *Leptolyngbya*-like sp. LEGE13412 and *N. antarctica* LEGE13457 seem promising for further exploitation in the framework of psoriasis, due to their antioxidant, anti-inflammatory and antiproliferative potential.

## 1. Introduction

Throughout history, nature has been acknowledged as a primordial source of bioactive molecules, being able to meet human demands for the prevention and treatment of the most varied diseases. In this regard, higher plants have been explored for centuries, with dozens of species in well-recognized use in traditional medicine, and several plant-derived molecules being the basis of many pharmaceutical formulations that are actually marketed [1]. This overexploitation of plant sources has, however, lead to a pseudo-stagnation in the discovery of new bioactive compounds of medical interest, causing the scientific community to extend their field into a broader spectrum of environments and organisms. Of them, microbial sources have become the main focus of pharmaceutical discovery in recent decades [2,3]. Microorganisms had been responsible for producing a huge number of bioactive compounds of medical interest, and stand out as an emerging source of profitable secondary metabolites. A significant percentage of the antibiotics and other bioactive molecules obtained so far have been produced by actinomycetes, fungi and bacteria; however, these are still far from answering human needs and pharmaceutical demands. The number of drugs reaching the market is still scarce, and there is low investment in new therapeutics for a significant number of diseases [4].

Psoriasis can be seen as a classic case of low therapeutic diversity. This painful and disabling disease affects around 125 million people worldwide and, besides having a huge negative impact on a patient’s quality of life, it has high treatment-associated costs that amount to 10 billion Euros annually, with work loss accounting for 40% of the cost burden [5]. For these reasons, and considering the limitations of the antipsoriatic formulations currently marketed, it is urgent that new effective therapeutic alternatives, microorganisms appearing as promising sources worth of exploitation [6]. Although the aetiology of psoriasis remains unclear, the disease is characterized by the presence of chronic inflammatory, itchy and erythematous skin lesions, with five recognized types (plaque, guttate, inverse, pustular and erythrodermic). Plaque is the most common form, appearing as raised and red patches covered with dead cells. It is often itchy and painful, with risk of cracking and bleeding [7]. Besides its physical form, the disease impacts patients’ mental health, and psoriatic patients can be exposed to social stigma and discrimination that often lead to a loss of self-esteem along with anxiety and depression [8]. The therapeutic choice to fight the symptoms is not always easy, and there are no marketed formulations that cover all the needs of this multifactorial disease. In this regard, the combination of topical and oral formulations is the most common approach. Topical and oral therapies aim to reduce inflammation and psoriatic plaques, with corticosteroids, calcipotriol, tazarotene, tar, anthralin and keratolytics being the most commonly prescribed. However, many side effects have been observed as a result of various drug combinations, most including antidepressants, which can increase the profile of adverse reactions, usually leading to therapeutic dropout [9]. In this sense, there is an urgent need for new multivalent therapeutic alternatives with less undesirable side effects and a favorable cost/benefit ratio, in order to improve quality of life among psoriatic patients and reduce their healthcare costs.

Well recognized for their enormous potential applications in pharmaceutical biotechnology, cyanobacteria appear as one of the most promising natural sources, with the capacity to produce a wide range of bioactive metabolites with diverse chemical structures [10]. Among them, carotenoids are particularly interesting, and have demonstrated the potential to fight the major symptoms of psoriasis and slow disease progression [11]. These compounds have revealed anti-inflammatory activity, suppressing nitric oxide (NO) production by lipopolysaccharide (LPS)-stimulated macrophage cells (RAW 264.7); this important biomarker is about 100 times higher in psoriatic plaques than in normal skin [12]. Carotenoids can also downregulate the expression of the inducible nitric oxide synthase (iNOS) and of cyclooxygenase 2 (COX-2), and they can reduce the release of tumor necrosis factor-α (TNF-α), interleukin (IL)-1β and IL-6 [13,14]. Carotenoids are also well recognized for their antioxidant activity, which is a real asset in the framework of psoriasis; through radical scavenging, carotenoids can reduce the exacerbation of inflammation, thus reducing tissue damage and accelerating repair [15]. Along with inflammation and oxidative stress, the resistance of keratinocytes to apoptosis may constitute one of the key pathogenic mechanisms in psoriasis [16]. Cyanobacteria-derived compounds have already shown their importance in this field, triggering apoptosis in different human and non-human cell lines through cell cycle arrest [17], mitochondrial dysfunction [18], alterations in caspase cascade [19], induction of p53 and Bax and inhibition of Bcl-2 expression [20] and alteration of membrane sodium channel dynamics [21], among other mechanisms.

This study aimed to exploit the biotechnological potential of different extracts of cyanobacteria of the LEGE Culture Collection (LEGE CC, http://lege.ciimar.up.pt/) (*Alkalinema aff. pantanalense* LEGE15481, *Cyanobium gracile* LEGE12431, *Nodosilinea (Leptolyngbya) antarctica* LEGE13457, *Cuspidothrix issatschenkoi* LEGE03282 and *Leptolyngbya*-like sp. LEGE13412) of CIIMAR in the topical treatment of psoriasis. We focused on the exploitation of the anti-inflammatory, antioxidant and antiproliferative potentials of these cyanobacteria, using different in vitro cell and cell-free assays. The extracts were chemically characterized for their pigment profiles, and a correlation between their chemical composition and biological activities was established. As far as we are aware, this is a pioneer study regarding both chemical characterization and evaluation of the mentioned bioactivities of the selected cyanobacteria strains.

## 2. Materials and Methods

### 2.1. Cyanobacteria Strains and Culturing Conditions

The isolates of the cyanobacteria strains used in this study (Table 1) were maintained in the LEGE CC (http://lege.ciimar.up.pt/), at the Interdisciplinary Centre of Marine and Environmental Research (CIIMAR/CIMAR).

Cyanobacteria biomass production was achieved through a 10-fold scale-up culture process (40 mL, 400 mL and 4 L). Terrestrial (*Nodosilinea (Leptolyngbya) antarctica* LEGE13457) and freshwater (*Alkalinema aff. pantanalense* LEGE15481, *Cyanobium gracile* LEGE12431 and *Cuspidothrix issatschenkoi* LEGE03282) strains were cultured in liquid Z8 medium while the marine strain (*Leptolyngbya*-like sp. LEGE13412) was cultured in the same medium supplemented with vitamin B12 and 25 g/L NaCl [22]. Growing conditions, at 25 °C, comprised a photoperiod of 16-/8-h light/dark cycles, respectively, with a light intensity of 10 mmol photons/s/m^2^. After the strains achieved proper growth (visually determined), the biomass was harvested by centrifugation (15,000 *g*, Sorvall ™ Bios 16 Bioprocessing Centrifuge, Thermo Fisher Scientific, Germany). Biomass resulting from the marine strain was washed with distilled water to remove salts. Fresh harvested biomass was freeze dried (LyoQuest ™, Telstar, Tokyo, Japan) and kept at −20 °C until chemical analysis and biological assaying.

### 2.2. Preparation of Cyanobacteria Extracts

Two different extracts (ethanol 70% *v/v* and acetone) were prepared from the dry biomass of each cyanobacteria strain. Briefly, 500 mg of dry biomass were suspended in 10 mL of the respective solvent and sonicated (Vibra-Cell ™ ultrasonic liquid processor, Sonics & Materials, INC., Newtown, USA) at a frequency of 70/80 Hz, for 3 min. The mixture was centrifuged (15,000 *g*, 10 min., 4 °C) (Gyrozen ™ 2236R, Vita Scientific, South Korea) to allow the sedimentation of all cell debris, and the supernatant was evaporated under reduced pressure (Buchi R-210 Rotavapor). The process was repeated five times, and the mixture was kept on ice and protected from light with aluminum foil, to avoid heating and oxidation of the extracts. The dried extracts were stored at −20 °C until further analysis.

### 2.3. Phytochemical Analysis

The determination of the carotenoid and chlorophyll profiles of the different cyanobacteria extracts was achieved by High Performance Liquid Chromatography (HPLC) with photo diode-array (PDA) detection (Waters Alliance 2695 Separations Module, Waters Corporation, Milford, USA) following a method previously described with minor modifications [23]. The dried ethanol and acetone extracts were resuspended in ethanol (70%, v/v) or acetone to a final concentration of 10 or 5 mg/mL, respectively, prior to HPLC analysis. Twenty microliters of each extract were analyzed using a Purospher STAR RP-18 Endcapped (5 µm, 250 × 4 mm, Merck Millipore, Massachusetts, USA) chromatographic column, and kept at a constant temperature of 25 °C during the analysis using a column heater (Waters Corporation, Milford, USA). The mobile phase consisted of two solvents, ethyl acetate (A) and acetonitrile:water 9:1 (*v/v*) (B), which were used to install the following gradient: 100% B at 0 min, 40% B at 31 min, 40% B at 36 min, 0% B at 38 min, 0% B at 43 min and 100% B from 50 to 55 min. All the solvents used in the chromatographic analysis were filtered using a GH Polypro (GHP) Membrane Disk Filter with 0.2 µm porosity (Pall Corporation, New York, USA) and degasified prior to analysis. The flow rate was 1 mL/min. Data were processed using Empower 2 Software (Waters, Milford, USA), and spectral data from all peaks were collected in the 250−750 nm range.

The tentative identification of the compounds was carried out by comparing their UV-Vis spectra and retention times with those of authentic standards. Carotenoid and chlorophyll quantification were achieved by measuring the absorbances recorded on the chromatogram’s face to authentic standards (Extrasynthese, Genay, France and Sigma Aldrich, St. Louis, USA), at 450 nm. Calibration curves (Table 2) were obtained for five serial dilutions of standard solutions that were selected as representative of the range of compound concentrations in the samples. Zeaxanthin, lutein, echinenone, β-carotene and chlorophyll *a* were quantified with their respective authentic standards. The unidentified carotenoids were quantified as zeaxanthin, β-carotene derivatives and α-carotene derivative as β-carotene, and the unidentified chlorophylls and chlorophyll *a* derivatives as chlorophyll *a*.

### 2.4. Antioxidant Potential

The antioxidant potential of the ethanol and acetone extracts of the cyanobacteria under study was assessed through the superoxide radical anion (O_2_^•-^) scavenging assay, as previously described [24]. A range of extract dilutions were prepared in phosphate buffer (19 mM, pH 7.4). Briefly, 50 µL of each dilution was mixed with 50 µL of a solution of β-nicotinamide adenine dinucleotide reduced form (NADH) (166 µM) and 150 µL of nitrotetrazolium blue chloride (NBT) (43 µM). The reaction was induced by the addition of 50 µL of phenazine methosulphate (PMS) (2.7 µM), and the reduction rate of NBT was monitored in kinetic function at 562 nm for 2 min using a Synergy HT Multi detection microplate reader operated by GEN5 (Biotek, Germany). Scavenging activity was expressed as the percentage of O_2_^•-^ scavenging, relative to the control. At least three independent assays were performed in duplicate.

### 2.5. Cell Assays

The murine macrophage cell line RAW 264.7 and the human keratinocyte cell line HaCaT (American Type Culture Collection, ATCC), were selected as models for the determination of the anti-inflammatory and antiproliferative potential of the cyanobacteria extracts under study, respectively.

#### 2.5.1. Cell Culture and Treatments

RAW 264.7 and HaCaT cells were cultured in Dulbecco’s Modified Eagle Medium (DMEM, Roti-CELL) with glutamine, without pyruvate, supplemented with 10% (v/v) of inactivated fetal bovine serum (FBS) and 1% (v/v) penicillin-streptomycin (Penicillin 100 IU/L, Streptomycin 100 µL/mL) at 37 °C, in a humidified atmosphere containing 5% CO_2_. The culture medium was renewed every two days, and cell passages (scraping for RAW 264.7 and trypsinization for HaCaT) were made at about 80% confluence [25,26]. 

Prior to extract exposition, cells were seeded in 96-well plates, at a density of 3.5 × 104 cells/well for RAW 264.7 and 2.5 × 103 cell/well for HaCaT, then incubated for 24 h. Cyanobacteria extracts were prepared in DMEM, sterilized by filtration using a 0.22-µm pore membrane and stored at −20 °C until cell assaying. Extract serial dilutions (100, 50, 25, 12.5 and 6.25 µg dry extract/mL) were prepared in DMEM with 0.25% DMSO, established as the maximum DMSO concentration that did not interfere with the assays. 

#### 2.5.2. Anti-inflammatory Potential

Exposed to a harmful stimulus, macrophages overexpress iNOS, leading to an increase in the release of NO to the extracellular space. The anti-inflammatory potential of the cyanobacteria extracts was assessed through their capacity to reduce NO produced by RAW 264.7 macrophages upon lipopolysaccharide (LPS) stimulation, following a procedure previously proposed [25]. Briefly, after a 2-h pre-treatment with the extract serial dilutions (or vehicle), RAW 264.7 cells were stimulated with LPS (1 µg/mL) and further incubated for 22 h. The effect of the extracts on NO produced by RAW 264.7 cells was also evaluated in the absence of LPS in order to cross-out the direct effect of the extracts on cell stimulation and measure the levels of basal NO produced by untreated cells. After the incubation period, NO was measured in the culture medium through a Griess reaction. Briefly, 75 µL of Griess reagent (sulfanilamide 10 mg/mL and ethylenediamine 1 mg/mL, prepared in 2% H_3_PO_4_) (Sigma-Aldrich, St. Louis, USA) was mixed with 75 µL cell supernatant and incubated in the dark for 10 min. The absorbance of the reaction product was determined at 562 nm. Results were expressed as the percentage of NO vs. the untreated control. At least four independent assays were performed in duplicate.

##### Cytotoxicity to Macrophages

In order to determine the effective non-toxic extract concentrations, the cytotoxicity of the extracts was monitored through the 3-(4,5-dimethylthiazole-2-yl)-2,5-diphenyltetrazolium bromide (MTT) assay, following a methodology previously described [25]. The assay consisted of the reduction of the yellow MTT to insoluble purple formazan crystals by dehydrogenizing metabolically active cells. After the incubation period of 24 h, 100 µL of MTT solution (0.5 mg/mL), freshly prepared in DMEM at 37 °C, was added to each well and incubated at 37 °C for 45 min. After the incubation period, the supernatant was removed and the resulting formazan crystals were dissolved in 100 µL DMSO. The absorbance of the colored product was determined at 515 nm using a Synergy HT Multi-Detection microplate reader (Biotek, Germany) operated by GEN5 software. Cytotoxicity was expressed as the percentage of cell viability vs. the control (0.25% DMSO). At least four independent assays were carried out in duplicate.

#### 2.5.3. Effect on Keratinocyte Proliferation

Psoriatic skin is characterized by the presence of plaques originating in the abnormal proliferation and differentiation of keratinocytes. The potential of cyanobacteria extracts to slow keratinocyte proliferation was assessed by determining the viability of human keratinocyte cell line HaCaT after periods of 24 and 48 h exposure to the extracts, with minor modifications to a method previously described [26]. Briefly, after the incubation period, the supernatant was removed and 100 µL of MTT solution (0.5 mg/mL), freshly prepared in DMEM at 37 °C, were added to each well, with the cells further incubated at 37 °C for 3.5 h. After that, the culture medium was removed and the MTT assay was carried out as previously described (in Section Cytotoxicity to Macrophages). Cytotoxicity was expressed as the percentage of cell viability vs. the control. At least four independent assays were performed in duplicate. DMSO (20%) was used as a positive control.

### 2.6. Statistical Analysis

Statistical analysis was performed using IBM SPSS STATISTICS software, version 23.0, IBM Corporation, New York, USA (2015). Data were analyzed for normality and homogeneity of variances by Kolmogorov–Smirnov and Leven’s tests, then submitted to a one-way ANOVA using a Tukey’s HSD (honest significant difference) as a post-hoc test, or to an unpaired *t*-test. A Pearson correlation test was used to compare normalized expression data between the chemical profiles and biological activities of cyanobacteria extracts.

## 3. Results and Discussion

### 3.1. Phytochemical Analysis

**Figure 1:**
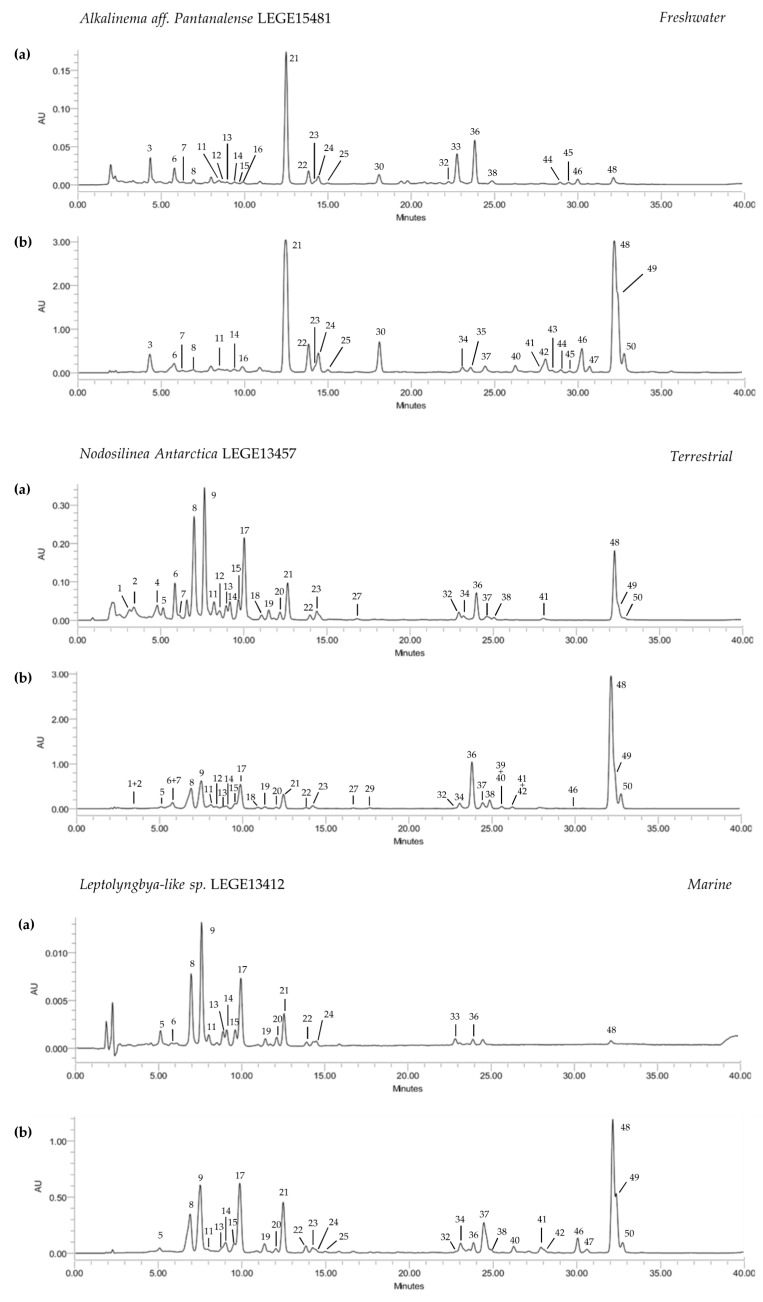
Carotenoid and chlorophyll profiles of ethanol (**a**) and acetone (**b**) extracts of cyanobacteria strains from different environments. HPLC-PDA recorded at 450 nm. Unidentified carotenoids (3, 5–8, 10–13, 15, 23, 25, 28, 31, 40–43), β-carotene oxygenated derivatives (9, 17, 34, 35), Lutein derivatives (14, 16, 19, 22, 24), Echinenone derivatives (30), Chlorophyll a derivatives (32, 33, 38), Unidentified Chlorophylls (44–47), Zeaxanthin (21), Canthaxanthin (26), Chlorophyll a (36), Echinenone (37), all-trans β-Carotene (48), α-Carotene derivative (49), 13-cis-β-Carotene (50).

The HPLC-PDA analysis of the ethanol and acetone extracts of the selected cyanobacteria strains allowed the tentative identification and quantification of 50 compounds, comprising eight chlorophylls and 42 carotenoids (Table 3). The compounds were analyzed over 35 min, with a good chromatographic resolution for both acetone and ethanol extracts (Figure 1). Acetone extracts were significantly richer in pigments, considering both carotenoids and chlorophylls (*p* < 0.05), than those obtained with ethanol, thus indicating acetone as the most effective solvent to meet the goals of the present study (Table 3, Figure 1). The terrestrial strain *N. antarctica* LEGE13457 presented the highest content in carotenoids (63.9 μg/mg, *p* < 0.05), but was closely followed by the freshwater strain *C. gracile* LEGE 12431 (57.8 μg/mg) (Table 3). The carotenoid content of the remaining cyanobacteria strains did not significantly differ (33.1–34.4 μg/mg, *p* > 0.05). Regarding chlorophylls, acetone extracts were richer than ethanol ones, with the amount varying between 73.2 μg/mg (*A. pantanalense* LEGE15481) and 417.6 μg/mg (*N. antarctica* LEGE 13457) (Table 3).

Chlorophylls were dominant over carotenoids, with chlorophyll *a* (36) and its derivatives (32, 33 and 38) being the most abundant and present in all strains. The predominance of these compounds in the terrestrial strain can be explained, at least in part, by the possible alteration of pigment production at the genetic level, which is caused by higher amounts of elements such as iron and phosphorous that are more abundant in these environments [27]. In fact, an incremental pigment production has already been reported for the diazotrophic cyanobacteria *Nostochopsis lobatus* upon supplementation with phosphorous and iron [28]. The alteration of chlorophyll production at the genetic level in terrestrial *N. antarctica* LEGE13457 can also be associated with rare earth oxide nanoparticles; for instance, it has been demonstrated that low cerium concentrations can act as catalysts in chlorophyll production [29]. Moreover, this strain originates from the Victoria Valley, Antarctica (Table 1), where the environment is harsh. This condition may regulate the metabolism of living organisms in order to increase the production of secondary metabolites with a key role in the maintenance of a balanced redox status, such as carotenoids. This deduction may also apply to *C. gracile* LEGE12431, the strain with second-highest pigment content, which originates from the Caburgua Lake, Chile (Table 1), an area that is also subjected to significant atmospheric fluctuations. The remaining strains, came from environments where the abiotic conditions do not fluctuate nor are they as severe as those pointed for *N. antarctica* LEGE13457 and *C. gracile* LEGE12431.

Zeaxanthin (21), lutein derivatives (14, 16, 19, 22 and 24) and echinenone (37) were the predominant xanthophylls, being present in almost all cyanobacteria strains, with *C. gracile* LEGE12431 accounting with the highest amounts (Table 3). Cantaxanthin (26) was only tentatively identified in *C. issatschenkoi* LEGE03282, which was among the strains with the highest concentration of xanthophylls. Echinenone was found in a significantly higher content in the marine strain *Leptolyngbya*-like sp. LEGE13412 (17.2 μg/mg, *p* < 0.05) (Figure 1), and had the most concentrated xanthophyll content. Carotenes were found in lower numbers than xanthophylls, which appeared in a significantly higher quantity (Table 3, Figure 1). All-*trans-β*-carotene (48) was the most abundant compound, followed by an α-carotene derivative (49) and its isomer 13-*cis*-*β*-carotene (50), with the terrestrial *N. antarctica* LEGE13457 displaying the highest content (27.7 μg/mg, *p* < 0.05), followed by the freshwater strain *C. gracile* LEGE12431 (24.0 μg/mg) (Table 3).

Despite the high biotechnological potential of pigments, their exploitation and profiling in cyanobacteria is still scarce. Works devoted to the analysis of pigments in cyanobacteria extracts have mainly focused on carotenoid biosynthesis and the influence of radiation in pigment production, and are limited mostly to the last two decades. Steiger et al. [30] investigated the light-dependent regulation of carotenoids in a strain of the genus *Synechocystis*, focusing the study on the major compounds (*β*-carotene, zeaxanthin and echinenone), and these compounds are in agreement with the major compounds found for the cyanobacteria strains analyzed herein (Table 3). This genus has also been explored by Paliwal et al., who made a nice approach to identify the effects of nutrients and salinity in carotenoid production in an attempt to make a targeted production of specific compounds [31].

Palinska et al. have attempted to analyze the carotenoid production patterns of the genera *Phormidium*, *Oscillatoria* and *Leptolyngbya* [32]. The authors concluded that, in a general way, strains from the same genera present identical pigment patterns, with *β*-carotene and zeaxanthin being the most common. Regarding the species belonging to the genus *Leptolyngbya*, a profile dominated by *β*-carotene and zeaxanthin, the presence of echinenone and cantaxanthin were reported in variable amounts according to different strains. These qualitative results are in accordance with those obtained herein, with the exception of echinenone, which was the major xanthophyll of *Leptolyngbya*-like sp. LEGE13412. Other than this, the carotenoid profiles of different strains of *Nodosilinea* and *Cyanobium* have recently been presented by Bavini et al. [26]. Likewise, *Nodosilinea* and *Cyanobium* were among the species with the highest carotenoid contents in our study, with *β*-carotene as the major compound. The main difference herein regards echinenone, which was not detected in the strains *Nodosilinea nodulosa* LEGE06102, *Cyanobium* sp. LEGE06113 and *Cyanobium* sp. LEGE07175 analyzed by the authors, and represents the major xanthophyll source found by us for these genera. This can be explained by the solvents used in extract preparation, as acetone was determined to be much more effective than ethanol 70% in the extraction of these compounds.

Hashtroudi et al. performed a study focused on the analysis of carotenoids of high nutritional significance (such as β-carotene, lycopene, lutein and zeaxanthin) in cyanobacteria belonging to the genera *Anabaena* and *Nostoc* isolated from terrestrial and aquatic ecosystems [33]. The extracts, obtained using a process involving ultrasonication and analyzed by HPLC, displayed large amounts of lycopene (up to 24,570 μg/g) and β-carotene (up to 8133 μg/g), which is comparable with the best natural sources of β-carotene. Our results are comparable to those of Hashtroudi et al., as we found significant amounts of β-carotene, reaching 27,695 μg/g in *N. antarctica* LEGE13457 and 23,963 μg/g in *C. gracile* LEGE12431. Thus, the cyanobacteria species explored herein are among the best natural sources of this carotene. Likewise, 13-*cis*-isomer was also detected herein, as well as diverse xanthophylls, which are recognized for their health benefits. Based on these analyses, we can assume the effectiveness of our methodology, as well as the effectiveness of the selected extraction solvent (acetone) for pigment extraction from the cyanobacteria biomass. Additionally, we can isolate the biotechnological potential of some cyanobacteria strains of the LEGE CC as promising sources of carotenoids of interest to human health. To the best of our knowledge, this is the first report on the pigment profile of these species.

### 3.2. Antioxidant Potential

The antioxidant potentials of the different cyanobacteria extracts were determined by evaluating their capacities to scavenge the physiologic radical O_2_^•-^ using an in vitro cell-free assay, and the results of the calculated inhibitory concentration (IC) values are presented in Table 4.

Superoxide is a by-product of oxygenic respiration with a central role in physiological processes; its accumulation leads to the deregulation of redox homeostasis and the production of different deleterious reactive oxygen species (ROSs), such as hydrogen peroxide (H_2_O_2_), hydroxyl radicals (OH^•^), hypochlorous acid (HOCl), and peroxynitrite (ONO_2_^•-^), which are in the basis of lipid peroxidation and damage proteins and DNA [34]. For this reason, evaluating the capacities of O_2_^•-^ inactivation via scavenging is growing in importance as a means to exploit the biotechnological potentials of natural extracts in pursuit of managing a wide variety of diseases.

As can be seen through in Table 4, acetone extracts were more effective than ethanol towards radical scavenging. Of the ethanol extracts with a radical scavenging capacity, only half reached IC_50_; however, this occurred at a concentration close to or surpassing 1 mg/mL. The ethanol extract from *C. issatschenkoi* LEGE03282 was the most effective, reaching IC_50_ at the lowest concentration tested (0.728 mg/mL, *p* < 0.05). Acetone extracts were much more interesting in terms of radical scavenging, with the species *C. issatschenkoi* LEGE03282 and *N. antarctica* LEGE13457 presenting the lowest IC_50_ values (0.286 and 0.319 mg/mL, respectively), followed by *A. pantanalense* LEGE15481 (0.382 mg/mL, *p* < 0.05). In an attempt to establish a relationship between the chemical profile and the radical scavenging capacity of the extracts, a Pearson correlation was performed. A negative correlation was observed between the total carotenoid content and IC values (−0.758, *p* < 0.05), emphasizing the contribution of these compounds to radical scavenging. However, this does not completely explain the results obtained (Table 4) for *C. gracile* LEGE12431, for example, which was one of the strains with the highest content in carotenoids but did not reach IC_50_ in O_2_^•-^ scavenging (max. of 43% scavenging at 1.67 mg/mL, data not shown). On the other hand, *C. issatschenkoi* LEGE03282 presented the best antioxidant potential albeit having a lower carotenoid content. A more detailed analysis revealed a strong correlation (−1.000, *p* < 0.01) between the radical scavenging capacity of this strain and its content in cantaxanthin. In fact, *C. issatschenkoi* LEGE03282 was the unique cyanobacteria strain presenting cantaxanthin, which may be responsible for the low IC values obtained for both ethanol and acetone extracts. On the other hand, despite having one of the highest contents of carotenoids, the acetone extract from C. gracile LEGE12431 only reached IC_25_, demonstrating the importance of chemical profiles for radical scavenging, and the different contributions of carotenoids depending on their chemical features. Indeed, the reaction between carotenoids and O_2_^•-^ is very unique. O_2_^•-^ inverts the direction of electron transfer when compared to other ROSs and, therefore, the reactivity between carotenoids and O_2_^•-^ greatly differs [35]. Even though the antioxidant capacities of carotenoids grow with increasing numbers of conjugated double bonds and decrease in the presence of hydroxy and keto groups [36], this behavior is not observed for O_2_^•-^, which shows the important role the nature of the reacting free radical plays in scavenging mechanisms [35]. For instance, Amaro et al. evaluated the radical scavenging capacity of an acetone extract of *Gloeothece* sp. against different free radicals and obtained better IC_50_ values for ABTS^+^ and ^•^NO than for 2,2-diphenyl-1-picrylhydrazyl (DPPH^•^) and O_2_^•-^ [37]. At the same time, other compounds present in the extracts with radical scavenging capacities, such as phenols, may have contributed to the IC values observed.

Bavini et al. have previously evaluated the radical scavenging capacities of ethanol extracts from different cyanobacteria strains [26]. The species of the genus *Cyanobium* (*Cyanobium* sp. LEGE06113 and *Cyanobium* sp. LEGE07175) and *Nodosilinea* (*Nodosilinea nodulosa* LEGE06102) analyzed by those authors did not display a capacity to scavenge O_2_^•-^ but only DPPH^•^, with the latter being more effective. This is in accordance with the results obtained herein. Differently, we reached IC_25_ in O_2_^•-^ scavenging using ethanol extracts, which leads us to believe that the biological activity of these microorganisms is more related to species-specific differences than to gender similarity. The same species have already been evaluated for their radical scavenging capacities by Anna et al. [38], but using a dichloromethane:methanol (2:1, *v/v*) extract. Those authors did not report IC values, but they found that: *Cyanobium* sp. LEGE06113 had a maximum scavenging of 19.5% for DPPH^•^ scavenging at 100 µg/mL, with no activity for O_2_^•-^; *Cyanobium* sp. LEGE07175 exhibited a maximum of 20.4% for DPPH^•^ scavenging at 100 µg/mL, and 47.39% for O_2_^•-^ at 10 µg/mL; and *Nodosilinea nodulosa* LEGE06102 displayed 12.2% for DPPH^•^ scavenging at 100 µg/mL and 28.9% for O_2_^•-^ at 10 µg/mL. Since different solvents were used in the extraction process, the results cannot be directly compared; however, the influence of species-specific features in the biological activity was again perceived.

Patias et al. [39] have also reported a correlation between carotenoid profiles and the antioxidant potential of extracts obtained from different cyanobacteria of the genus *Aphanothece*, *Chlorella* and *Scenedesmus*, inferring that samples richer in highly unsaturated carotenoids are more effective as radical scavengers. Aside from this, we have noted that chlorophylls may also contribute to O_2_^•-^ scavenging. For instance, *A. pantanalense* LEGE15481 and *C. issatschenkoi* LEGE03282 presented significantly lower contents in highly unsaturated carotenoids than *N. antarctica* LEGE13457 (Table 3), but similar IC_50_ (Table 4). On the other hand, these strains were characterized by a high content in chlorophylls 44–47, which were strongly correlated with the scavenging activity of the extracts (−0.927, *p* < 0.01). In fact, the capability of chlorophylls and their derivatives to scavenge free radicals and prevent the formation of reactive species has already been reported [40]. Altogether, analysis of whole pigment profiles of cyanobacteria extracts seems to be valuable in predicting their antioxidant potentials and, consequently, to inferring their biotechnological potential.

### 3.3. Anti-Inflammatory Potential

Inflammation is a process whereby the host responds to harmful stimuli with the aim of replacing organism homeostasis and promoting tissue repair. This complex and multifactorial process involves a wide array of immune cells and mediators that communicate in a multifactorial network. The disruption of a normal inflammatory process results in the inability of an organism to replace homeostasis, leading to a cyclic process with a constant production of inflammatory mediators and the establishment of a chronic framework [41]. Psoriasis is a chronic inflammatory disease characterized by hyperplasia, dilatation and the proliferation of dermal blood vessels. It is associated with an accumulation of inflammatory cells and the constant overproduction of inflammatory mediators that are associated with a wide range of co-morbidities involving metabolic diseases and psychological disorders [42]. In this sense, targeting inflammation constitutes one of the first and most comprehensive measures in restoring balance and reducing tissue damage. Although a wide array of cells and mediators are involved in the onset of chronic inflammation, some of them can be pointed out. In this survey, we have focused on exploiting the effects of cyanobacteria extracts on the metabolism of macrophages. These cells play a pivotal role in the entire inflammatory process and are responsible for the production of different inflammatory cytokines such as IL-1β, IL-6 and TNF-α, as well as inflammatory mediators such as NO, and modulation can prevent the onset and progression of inflammation. In fact, NO is about 100 times higher in psoriatic plaques (which actively produce NO) when compared to normal skin [12]. This overproduction of NO in psoriatic lesions induces keratinocyte hyperproliferation, enhancing the importance of radical scavengers and iNOS inhibitors. RAW 264.7 cells are a well-established model for predicting the anti-inflammatory potential of drugs, since they easily increase NO production upon stimulation through incremental iNOS expression. This model has been widely used in the context of psoriasis [43,44] and was adopted in this study.

**Figure 2:**
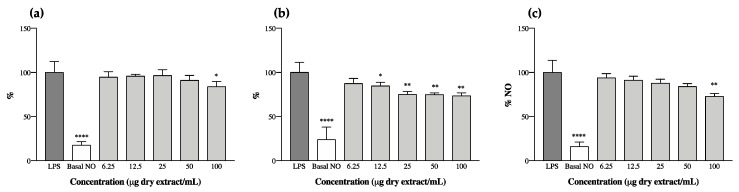
Nitric oxide (NO) production by RAW 264.7 cells in the presence of cyanobacteria acetone extracts after stimulation with lipopolysaccharide (LPS). (**a**) *Alkalinema aff. pantanalense* LEGE15481, (**b**) *Nodosilinea (Leptolyngbya) antarctica* LEGE13457 and (**c**) *Leptolyngbya*-like sp. LEGE13412. Results are expressed as % of NO relative to the control stimulated with LPS. Basal NO represents the NO produced by RAW 264.7 cells without LPS stimulation. Results are expressed as the mean ± SD of at least four independent assays, performed in duplicate. * *p* < 0.05, ** *p* < 0.01, **** *p* < 0.0001 (ANOVA, Tukey HSD).

The anti-inflammatory potential of cyanobaceria extracts was explored through the analysis of their effect on the levels of NO produced by RAW 264.7 macrophages upon LPS stimulation. We observed that ethanol and acetone extracts had an opposite behavior to one another—all ethanol extracts increased the levels of NO produced by RAW 264.7 on their own, suggesting a pro-inflammatory potential (Figure S1) and thus not meeting the aim of our study. This phenomena could have occurred, at least in part, as a result of the presence of cyanobacteria endotoxins (LPS), as these molecules are more prone to being extracted from more polar solvent mixtures (e.g., ethanol 70% *v/v*), causing macrophage activation through interactions with Toll-like receptors (TLR4) present in cell membranes [45]. Of the acetone extracts, those of *C. issatschenkoi* LEGE03282 and *C. gracile* LEGE12431 also presented pro-inflammatory behavior (Figure S2), and only three were able to reduce the levels of NO after LPS stimulation (Figure 2). It is worth mentioning, however, that with the exception of *C. issatschenkoi* LEGE03282 ethanol extract, no cytotoxicity was observed for the macrophage cell line under the tested concentrations (Figure S3).

*N. antarctica* LEGE13457 was the most effective, reaching an IC_25_ of 22.2 ± 1.6 µg/mL, followed by *Leptolyngbya*-like sp. LEGE13412, with an IC_25_ of 84.1 ± 8.4 µg/mL. *Alkalinema aff. pantanalense* LEGE15481 did not reach IC_25_, presenting a maximum NO reduction of 18% at the highest concentration tested (100 µg/mL).

To date, few studies have addressed the anti-inflammatory potential of cyanobacteria extracts for the purpose of biotechnological application, with the majority of the studies having been devoted to isolated compounds. The study undertaken by Gomes et al. [46] can, to some extent, be used for comparison in regard to the screening of cyanobacteria strains with anti-inflammatory potential. Those authors explored the anti-inflammatory potential of fractions of different polarities obtained from different cyanobacteria strains, using a similar methodology of LPS-stimulated RAW 264.7 macrophages. The strains explored by the authors included two *Leptolyngbya* (*Leptolyngbya* sp. LEGE07075 and *Leptolyngbya* sp. LEGE07084) and one *Cyanobium* (*Cyanobium* sp. LEGE07175), which share the same genus as two of the strains explored herein. The authors did not report anti-inflammatory potential for *Cyanobium* sp. and did not consider the results obtained with *Leptolyngbya* to be very promising, which, even using different extracts, is in agreement with the results obtained by us. Additionally, those authors also reported the pro-inflammatory behavior of some cyanobacteria strains, corroborating our findings for all ethanol (S1) and for two of the acetone extracts (Figure S2) analyzed.

The decrease in NO levels observed herein (Figure 2) can be correlated with the presence of individual compounds in the extracts, or to a synergism between the whole pigments. For instance, compounds 8, 11 and 23 were predominant or unique in the strains that displayed anti-inflammatory potential; others, like carotenes and echinenone, were dominant in *N. antarctica* LEGE13457 and *Leptolyngbya*-like sp. LEGE13412, which were the most promising strains (Figure 2b,c and Table 3).

It has been previously reported that lutein can decrease NO production in LPS-stimulated RAW 264.7 cells through the reduction of iNOS expression at the mRNA level, suggesting the anti-inflammatory properties of this xanthophyll [47]. In fact, both lutein (20) and its derivatives (14, 16, 19, 22 and 24) were found in the cyanobacteria extracts under study. However, the highest content in lutein did not mean a higher anti-inflammatory potential. Murakami et al. [48] conducted a very interesting study wherein they screened the capacities of different carotenoids to modify NO generation in RAW 264.7 cells, and made a structure/activity relationship approach. The authors observed that the insertion of a single hydroxyl group into the β-end group of β-carotene led to an increase in activity, whereas the introduction of two hydroxyl groups into both β-ends resulted in a loss of activity (e.g., zeaxanthin and lutein). In this regard, it comes as no surprise that *A. pantanalense* LEGE15481 presented the lowest activity among the bioactive strains, once it was the one with highest content in zeaxanthin (Table 3, Figure 2a). Similarly, the pro-inflammatory activity of *C. gracile* LEGE12431 (Figure S2) can be explained, at least in part, by the high content of zeaxanthin and lutein derivatives present in the extract (Table 3). The authors of [48] also reported that carotenoids presenting a 4-oxo-β-end group in their structure (e.g., cantaxanthin) were responsible for enhancing NO production. This could explain the pro-inflammatory activity obtained for *C. issatschenkoi* LEGE03282 (Figure S2), as this was the only strain presenting cantaxanthin and had one of the highest contents of xanthophylls from those profiled in this study (Table 3). In fact, cantaxanthin had already been associated with an increase in mitogen-induced lymphocyte proliferation [49], with an enhancement in the release of IL-1β and TNF-α [50] and with an enhanced expression of activation markers in human peripheral blood mononuclear cells in vitro [51].

There is no doubt about the beneficial effect of carotenoids in patients with psoriasis, with low levels having already been correlated with a prevalence of the disease [52]. However, it has been proven herein that carotenoid profiles play a key role in the bioactivity of extracts. The HPLC-PDA methodology employed herein proved to be a valuable tool for pigment profiling, allowing the prediction of the antioxidant and anti-inflammatory potential (and, consequently, biotechnological value) of cyanobacteria extracts through the careful analysis of their elementary compositions. Going against the aim of the present study, the acetone extracts of *N. antarctica* LEGE13457 and *Leptolyngbya*-like sp. LEGE13412 seem biotechnologically interesting since, beyond presenting anti-inflammatory potential, they were among the strains with the best capacity to scavenge the physiological free radical O_2_^•-^, suggesting a multifactorial mode of action that could be an asset in the framework of psoriasis.

### 3.4. Antiproliferative Potential

Keratinocytes are part of a dynamic interplay among various cells, playing a central role in the pathogenesis of psoriasis. Their hyperproliferation and abnormal differentiation is central to the formation of psoriatic plaque that, besides having a huge negative impact on an individual’s appearance and self-esteem, is limiting due to a provocation of itching and pain. Many anti-psoriatic therapies have focused on chemically removing or arresting keratinocyte proliferation, aiming skin thickening and establishment a normal epidermis recovery, in order to avoid epidermal hyperplasia [53].

**Figure 3:**
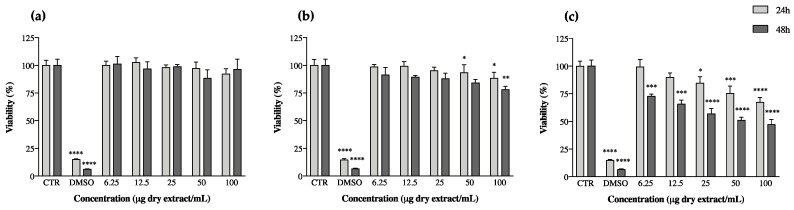
Keratinocyte (HaCaT) viability after 24 and 48 h of incubation with cyanobacteria acetone extracts. (**a**) *Alkalinema aff. pantanalense* LEGE15481, (**b**) *Nodosilinea (Leptolyngbya) antarctica* LEGE13457 and (**c**) *Leptolyngbya*-like sp. LEGE13412. Results are expressed as % of MTT reduction vs. the untreated control (CTR). DMSO (20%) represents the positive control. Results are expressed as the mean ± SD of at least four independent assays, performed in duplicate. * *p* < 0.05, ** *p* < 0.01, *** *p* < 0.001, **** *p* < 0.0001 (ANOVA, Tukey HSD).

Beyond this, keratinocytes also produce TNF-α, which stimulates the transformation of macrophages into dendritic cells, which are among the main players in the establishment of chronic inflammation [54]. Following in this direction, the effect of cyanobacteria extracts on keratinocyte proliferation was assessed by measuring their effects on cell viability after periods of 24 and 48 h of exposition to the extracts through the MTT assay (Figure 3). Almost all ethanol extracts reduced keratinocyte viability (Figure S4); however, once they had previously presented pro-inflammatory potential (point 3.3), they were not considered promising for the purpose of our study (Figure S1). This behavior has already been reported by Bavini et al. [26], who observed a decrease in keratinocyte viability when exposed to ethanol extracts of the cyanobacteria *Phormidium* sp. LEGE05292 and Oscillatoriales LEGE07167. The acetone extracts of *C. issatschenkoi* LEGE03282 and *C. gracile* LEGE12431 were also promising towards the slowing of keratinocyte proliferation (Figure S5), but, like ethanol extracts, they too revealed pro-inflammatory behavior (Figure S2). The results of the most promising extracts, considering our previous results and going against the goal of our study, are shown in Figure 3.

*A. pantanalense* LEGE15481 did not show toxicity to HaCaT, and *N. antarctica* LEGE13457 presented a maximum reduction in keratinocyte viability of ca. 12% for the highest concentration tested (100 µg/mL, *p* < 0.05) after a period of 48 h exposure to the extract. *Leptolyngbya*-like sp. LEGE13412 was the most promising strain in this assay, showing a dose-dependent activity at both 24 and 48 h of exposition; the reduction in keratinocyte viability reached its maximum after 48 h, corresponding to a decrease in viability of approximately 50% (*p* < 0.05) (Figure 3).

The HaCaT cell model has been widely used in this thematic, with the viability assay being set as a good indicator for the screening of the antipsoriatic potential of different extracts; for instance, Tse et al. have already confirmed the antipsoriatic activity of Chinese herbs used in traditional medicine for treating psoriasis via the same methodology as the one herein [53]. Regarding cyanobacteria, the studies in this thematic are still scarce and have not yet directly explored the biotechnological potential of extracts. For instance, Gudmundsdottir et al. explored the antipsoriatic potential of exopolysaccharides extracted from *Cyanobacterium aponinum*, a strain present in the Blue Lagoon in Iceland, where regular baths had been associated with an amelioration of psoriatic symptoms [55,56]. Among others, the authors reported a reduction in the secretion of chemokines implicated in the recruitment of inflammatory cells by keratinocytes. Furthermore, it has been hypothesized that high concentrations of arsenic in the water could induce keratinocyte apoptosis in psoriatic lesions, improving skin conditions [57]. This assumption strengthens our results, as the extracts evaluated herein reduced the viability of HaCaT, which demonstrates their potential to improve psoriatic conditions through a similar mechanism to that hypothesized previously.

Carotenoids may act as antioxidants or as pro-oxidants, depending on their concentration in cells, as well as in cell oxidative environments, with high concentrations being linked to the enhancement of pro-oxidant effects in biological systems. In fact, some studies have explored the role of carotenoids in cell-growth modulation. β-carotene and cantaxanthin have been reported to reduce the proliferation of human keratinocytes [58], and it has also been observed that β-carotene decreases the expression of Bcl-2, a pro-oncogene product involved in protecting cells from apoptosis [59]. β-carotene has been reported as possibly able to modulate the redox-sensitive molecular pathways involved in cell proliferation and apoptosis [60]. This hypothesis of a pro-oxidant role was supported by a study wherein a combination of carotenoids with other antioxidants in an animal model increased the inhibition of cancer incidence and/or mortality than occurred through the administration of carotenoids alone [61]. This emphasizes the importance of exploring bioactive extracts from a biotechnological point of view, as the variability and profiles of metabolites can act in synergy to originate a polyvalent product with multifactorial modes of action, thus covering the major needs of patients with psoriasis without the need of polymedication and, consequently, avoiding the common framework of adverse reactions.

## 4. Conclusions

Cyanobacteria are extremely interesting organisms that should be at the forefront of drug discovery, not only for their capability to synthesize a wide range of bioactive compounds, but also for allowing an environmentally friendly biomass production without damaging ecosystems or interfering with biodiversity. The present work is a pioneering study regarding the pigment profiling of the selected cyanobacteria species and the exploitation of the biotechnological potential of their bioactive extracts in the treatment of psoriasis. Two of the analyzed strains, in particular, revealed potential for being applied in the development of a topical galenic for the treatment of psoriasis: *N. antarctica* LEGE13457 and *Leptolyngbya*-like sp. LEGE13412 presented a chemical profile that met all the necessary requirements for fighting the major signs and symptoms of psoriasis. In sum, the acetone extracts of these two strains revealed a capacity to scavenge deleterious physiological free radicals, an anti-inflammatory potential in stimulated macrophages, an ability to induce apoptosis in HaCaT and a differential toxicity, according to the cell line under study. Although the mechanisms driving these factors are still not completely elucidated, this work represents a giant step towards the exploitation of cyanobacteria from a health-allied biotechnological point of view. Moreover, our work opens doors for future testing in animal models in order to test the effectiveness of the extracts in vivo.

## Figures and Tables

**Table 1:** Origin and morphological features of the cyanobacteria strains used in the study ^1^.

Genus/Strain	LEGECC Code	Origin/Environment
*Alkalinema aff. pantanalense*	LEGE15481	Amazon River, Macapá—Brazil Freshwater (Smooth Biofilm)
*Cyanobium gracile*	LEGE12431	Caburgua Lake, La Araucania Region—Chile Freshwater (Aggregate Forming)
*Nodosilinea (Leptolyngbya) antarctica*	LEGE13457	McMurdo Dry Valleys, Victoria Valley—Antarctica Terrestrial (Smooth Biofilm)
*Cuspidothrix issatschenkoi*	LEGE03282	Maranhão Dam Reservoir, Montargil—Portugal Freshwater (Homogenous Growth)
*Leptolyngbya-like* sp.	LEGE13412	Porto Metropolitan Area—Portugal Marine (Mucilaginous)

^1^ (LEGE CC, http://lege.ciimar.up.pt/).

**Table 2:** Calibration curves of carotenoid standards and chlorophyll *a*.

Standard	Calibration Curve	*r* ^2^	LOD ^a^	LOQ ^b^
Lutein	*y* = 141092914*x* + 5527	0.9998	0.0002867	0.0008688
Chlorophyll *a*	*y* = 7471178*x* + 2673	0.9998	0.0014721	0.0044608
Zeaxanthin	*y* = 1406075946*x* − 138160	0.9987	0.0003949	0.0011965
β-Carotene	*y* = 290231487*x* + 172758	0.9997	0.0019354	0.0058649
Echinenone	*y* = 61438587*x* + 12035	0.9995	0.0005080	0.001538
Neoxanthin	*y* = 219321360*x* + 57794	0.9999	0.0004293	0.0013009
Canthaxanthin	*y* = 5662994*x* − 2341	0.9994	0.0030490	0.0092395

^a^ limit of detection (mg/mL); ^b^ limit of quantification (mg/mL).

**Table 3:** Carotenoid and chlorophyll contents (µg/mg of dry extract) in ethanol 70% *v/v* and acetone extracts of cyanobacteria, determined by HPLC-PDA ^1,2^.

Peak	Compound	RT (min)	*Alkalinema aff. pantanalense* LEGE15481		*Cyanobium gracile* LEGE12431		*Nodosilinea (Leptolyngbya) antarctica* LEGE13457		*Cuspidothrix issatschenkoi* LEGE03282		*Leptolyngbya*-like sp. LEGE13412	
			Ethanol	Acetone	Ethanol	Acetone	Ethanol	Acetone	Ethanol	Acetone	Ethanol	Acetone
**1**	Unidentified Carotenoid	3.13	nd	nd	nd	nd	0.039 ± 0.006	0.093 ± 0.008 *	nd	nd	nd	nd
**2**	Unidentified Carotenoid	3.39	nd	nd	nd	nd	0.051 ± 0.008		nd	nd	nd	nd
**3**	Unidentified Carotenoid	4.32	0.033 ± 0.002 ^b^	0.187 ± 0.003 ^a^	nd	nd	nd	nd	nd	nd	nd	nd
**4**	Unidentified Carotenoid	4.79	nd	nd	nd	nd	0.053 ± 0.003	nd	nd	nd	nd	nd
**5**	Unidentified Carotenoid	5.15	nd	nd	nd	nd	0.034 ± 0.003 ^d^	0.225 ± 0.003 ^a^	0.014 ± <0.001 ^e^	0.069 ± 0.002 ^c^	0.011 ± <0.001 ^e^	0.110 ± 0.008 ^b^
**6**	Unidentified Carotenoid	5.68	0.026 ± 0.001 ^c^	0.100 ± <0.001 ^a^	nd	nd	0.080 ± <0.001 ^b^	0.451 ± 0.007 *	nd	nd	0.010 ± <0.001 ^c^	nd
**7**	Unidentified Carotenoid	6.28	0.012 ± 0.001 ^b^	0.055 ± <0.001 ^a^	nd	nd	0.011 ± <0.001		0.010 ± <0.001 ^c^	nd	nd	nd
**8**	Unidentified Carotenoid	7.01	0.013 ± <0.001 ^e^	0.050 ± <0.00 ^d^	nd	nd	0.251 ± 0.004 ^c^	1.298 ± 0.017 ^a^	0.011 ± <0.001 ^e^	0.058 ± <0.001 ^d^	0.014 ± <0.001 ^e^	0.439 ± 0.008 ^b^
**9**	β-Carotene oxygenated derivative	7.64	nd	nd	nq	nq	1.259 ± 0.011 ^c^	6.940 ± 0.118 ^a^	nd	nd	nq	3.004 ± <0.001 ^b^
**10**	Unidentified Carotenoid	7.75	nd	nd	0.011 ± <0.001 ^b^	nq	nd	nd	0.010 ± <0.001 ^c^	0.055 ± <0.001 ^a^	nd	nd
**11**	Unidentified Carotenoid	8.21	0.015 ± <0.001 ^e^	0.099 ± 0.001 ^b^	nd	nd	0.055 ± 0001 ^d^	0.269 ± <0.001 ^a^	nd	nd	0.010± <0.001 ^f^	0.080 ± 0.001 ^c^
**12**	Unidentified Carotenoid	8.55	0.012 ± <0.001 ^c^	nd	nd	nd	0.033 ± 0.001 ^b^	0.127 ± 0.001 ^a^	nd	nd	nd	nd
**13**	Unidentified Carotenoid	8.94	0.012 ± <0.001 ^a^	nd	nd	nd	0.039 ± <0.001 ^c^	0.120 ± 0.003 ^a^	nd	nd	0.011 ± <0.001 ^d^	0.061 ± 0.002 ^b^
**14**	Lutein Derivative	9.17	0.014 ± <0.001 ^e^	0.202 ± 0.014 ^d^	0.003 ± <0.001 ^e^	0.384 ± 0.011 ^c^	0.354 ± 0.019 ^c,d^	1.107 ± 0.002 ^a^	nd	nd	0.004 ± <0.001 ^e^	0.842 ± 0.126 ^b^
**15**	Unidentified Carotenoid	9.68	0.010 ± <0.001 ^d^	nd	nd	nd	0.054 ± 0.002 ^c^	0.225 ± 0006 ^a^	nd	nd	0.011 ± <0.001 ^d^	0.093 ± 0.010 ^b^
**16**	Lutein Derivative	9.87	0.019 ± <0.001 ^b^	0.394 ± 0.015 ^a^	nd	nd	nd	nd	nd	nd	nd	nd
**17**	β-Carotene oxygenated derivative	10.02	nd	nd	nq	1.102 ± 0.026 ^c^	0.865 ± 0.009 ^c^	5.589 ± 0.069 ^a^	nd	nd	nq	2.255 ± 0.183 ^b^
**18**	Unidentified Carotenoid	11.06	nd	nd	nd	nd	0.020 ± <0.001 ^b^	0.085 ± <0.001 ^a^	nd	nd	nd	nd
**19**	Lutein Derivative	11.50	nd	nq	nq	0.216 ± 0.007 ^c^	0.193 ± 0005 ^c^	0.826 ± 0.012 ^a^	nd	nd	nq	0.682 ± 0.040 ^b^
**20**	Lutein	12.19	nd	nd	nd	nd	0.152 ± 0005 ^c^	0.582 ± 0.010 ^a^	nd	nd	0.001 ± <0.001 ^d^	0.329 ± 0.022 ^b^
**21**	Zeaxanthin	12.64	0.146 ± 0.005 ^e^	2.348 ± 0.001 ^b^	0.033 ± 0.001 ^f,g^	6.299 ± 0.025 ^a^	0.088 ± 0.001 ^e,f^	0.625 ± 0.008 ^c^	nd	nd	0.012 ± <0.001 ^g^	0.384 ± 0.042 ^d^
**22**	Lutein Derivative	14.00	0.129 ± <0.001 ^c,d^	2.497 ± 0.268 ^b^	0.044 ± 0.001 ^d^	5.916 ± 0.055 ^a^	0.099 ± 0.001 ^c,d^	0.485 ± 0.007 ^c^	nd	nd	nq	0.444± 0.068 ^c,d^
**23**	Unidentified Carotenoid	14.40	0.012 ± <0.001 ^e^	0.107 ± 0.004 ^b^	nq	nq	0.004 ± <0.001 ^d^	0.170 ± 0.213 ^a^	nd	0.080 ± <0.001 ^c^	nd	0.066 ± 0.008 ^c^
**24**	Lutein Derivative	14.58	0.078 ± 0.001 ^c,d^	1.154 ± 0.071 ^b^	0.039 ± <0.001 ^d^	5.327 ± 0.121 ^a^	nd	nd	nd	nd	0.002 ± 0.002 ^d^	0.282 ± 0.040 ^c^
**25**	Unidentified Carotenoid	14.94	0.011 ± <0.001 ^e^	0.064 ± <0.001 ^b^	nd	0.028 ± <0.001 ^d^	nd	nd	nd	0.073 ± <0.001 ^a^	nd	0.032 ± <0.001 ^c^
**26**	Canthaxanthin	15.83	nd	nd	nd	nd	nd	nd	0.230 ± 0002 ^b^	9.300 ± 0.220 ^a^	nd	nd
**27**	Unidentified Carotenoid	16.81	nd	nd	nd	nd	0.013 ± <0.001 ^b^	0.052 ± <0.001 ^a^	nd	nd	nd	nd
**28**	Unidentified Carotenoid	18.32	nd	nd	nd	nd	nd	nd	nd	0.062 ± 0.001	nd	nd
**29**	Unidentified Carotenoid	18.53	nd	nd	nd	nd	nd	0.061 ± <0.001	nd	nd	nd	nd
**30**	Echinenone Derivative	18.72	0.249 ± 0.004 ^b^	5.797 ± 0.119 ^a^	nd	nd	nd	nd	nd	nd	nd	nd
**31**	Unidentified Carotenoid	20.18	nd	nd	nd	nd	nd	nd	nd	0.079 ± <0.001	nd	nd
**32**	Chlorophyll *a* Derivative	22.48	0.526 ± 0.005 ^d^	nd	0.147 ± 0.010 ^e^	nd	3.396 ± 0.019 ^a^	0.672 ± 0.020 ^c^	nd	nd	nd	1.084 ± 0.007 ^b^
**33**	Chlorophyll *a* Derivative	22.94	7.147 ± 0.044 ^b^	nd	0.502 ± 0.003 ^d^	4.466 ± 0.083 ^c^	nd	nd	0.475 ± 0.0103 ^d^	11.295 ± 0.374 ^a^	0.036 ± 0.017 ^d^	nd
**34**	β-Carotene Oxygenated Derivative	23.67	nd	0.043 ± 0.004 ^d^	nq	1.044 ± 0.021 ^b^	nq	1.251 ± 0.022 ^a^	nd	nq	nd	0.331 ± 0.042 ^c^
**35**	β-Carotene Oxygenated Derivative	23.79	nd	0.104 ± 0.002	nd	nd	nd	nd	nd	nq	nd	nd
**36**	Chlorophyll *a*	24.00	9.101 ± 0.030 ^e,f^	nd	3.257 ± 0.037 ^e,f^	325.047 ± 5.764	11.729 ± 0.034 ^e^	348.97 ± 4.190 ^a^	1.854 ± 0.005 ^e,f^	196.163 ± 4.469	0.031 ± 0.010 ^f^	63.636 ± 2.645 ^d^
**37**	Echinenone	24.61	nd	1.584 ± 0.037 ^c^	nq	7.313 ± 0.152 ^b^	0.300 ± 0.002 ^c^	6.481 ± 0.127 ^b^	0.060 ± <0.001 ^c^	nd	nd	17.188 ± 1.217 ^a^
**38**	Chlorophyll *a* Derivative	25.06	0.907 ± 0.011 ^c^	nd	0.420 ± 0.006 ^c^	67.075 ± 1.441 ^a^	1.010 ± 0.032 ^c^	65.127 ± 0.957 ^a^	0.464 ± 0.005 ^c^	nd	nd	15.273 ± 1.314 ^b^
**39**	Unidentified Carotenoid	25.59	nd	nd	nd	nd	nd	0.126 ± 0.002	nd	nd	nd	nd
**40**	Unidentified Carotenoid	26.19	nd	0.106 ± 0.001 ^b^	nd	0.081 ± 0.001 ^d^	nd	0.092 ± 0.002 ^c^	nd	0.112 ± 0.001 ^a^	nd	0.041 ± <0.001 ^e^
**41**	Unidentified Carotenoid	27.92	nd	0.121 ± 0.003 ^a^	nd	0.116 ± 0.004 ^a^	0.015 ± <0.001 ^c^	0.079 ± 0.002 ^b^	nd	0.224 ± 0.005 *	nd	0.114 ± 0.007 *
**42**	Unidentified Carotenoid	28.67	nd	0.207 ± <0.001 ^a^	nd	nd	nd	0.045 ± 0.001 ^b^	nd		nd	
**43**	Unidentified Carotenoid	29.07	nd	0.078 ± 0.001	nd	nd	nd	nd	nd	0.077 ± <0.001	nd	nd
**44**	Unidentified Chlorophyll	29.51	0.391 ± 0.013 ^c^	5.162 ± 0.081 ^a^	nd	nd	nd	nd	nq	2.619 ± 0.034 ^b^	nd	nd
**45**	Unidentified Chlorophyll	30.03	0.342 ± 0.005 ^c^	2.100 ± 0.033 ^a^	nd	nd	nd	nd	0.004 ± 0.007 ^d^	1.794 ± 0.013 ^b^	nd	nd
**46**	Unidentified Chlorophyll	30.61	1.016 ± 0.012 ^d,e^	54.138 ± 0.949 ^a^	nd	nd	nd	2.809 ± 0.053 ^d^	0.279 ± <0.001 ^e^	26.106 ± 0.584 ^b^	nd	8.120 ± 0.435 ^c^
**47**	Unidentified Chlorophyll	31.19	nd	11.802 ± 0.227 ^a^	nd	nd	nd	nd	0.007 ± 0.006.^d^	10.198 ± 0.171 ^b^	nd	2.370 ± 0.042 ^c^
**48**	All-*trans* β-Carotene	32.32	nq	15.196 ± 0.779 ^c^	nq	23.963 ± 0.181 ^b^	0.859 ± 0.021 ^f^	27.695 ± 0.337 ^a^	nq	12.384 ± 0.249 ^d^	nq	5.762 ± 0.389 ^e^
**49**	α-Carotene derivative	32.89	nd	1.624 ± 0.289 ^d^	nd	4.164 ± 0.257 ^c^	0.060± 0.010 ^e^	5.940 ± 0.030 ^b^	nq	10.135 ± 0.349 ^a^	nd	0.684 ± 0.141 ^e^
**50**	13-*cis*-β-Carotene	33.37	nd	1.076 ± 0.0310 ^c^	nq	1.874 ± 0.054 ^b^	nq	2.884 ± 0.044 ^a^	nd	1.708 ± 0.066 ^b^	nd	0.371 ± 0.032 ^d^
**Total Carotenoids**			0.791 ± 0.006 ^e^	33.193 ± 0.390 ^c^	0.131 ± 0.001 ^e^	57.829 ± 0.400 ^b^	5.012 ± 0.113 ^d^	63.926 ± 0.839 ^a^	0.335 ± 0.01 ^e^	34.415 ± 0.897 ^c^	0.085 ± 0.007 ^e^	33.594 ± 2.204 ^c^
**Total Chlorophylls**			19.430 ± 0.060 ^f^	73.202 ± 1.290 ^e^	4.219 ± 0.047 ^g,h^	396.588 ± 7.289	16.134 ± 0.047	417.576 ± 5.221	3.083 ± 0.019 ^g,h^	248.175 ± 5.645	0.067 ± 0.027 ^h^	90.481 ± 4.429 ^d^

^1^ Values are expressed as mean ± SD of two determinations; ^2^ nd, not detected; nq, not quantified; RT, retention time; * Co-elution; different superscript letters in the same row denote statistical differences at *p* < 0.05 (ANOVA, Tukey HSD/unpaired *t*-test).

**Table 4:** Inhibitory concentration (IC) values (mg of dry extract/mL) assessed for the O_2_^•-^ scavenging assay of the cyanobacteria ethanol 70% *v/v* and acetone extracts ^1,2^.

Species	IC_25_		IC_50_	
	Etanol	Acetone	Etanol	Acetone
*Alkalinema aff. Pantanalense* LEGE15481	0.657 ± 0.041 ^b^	0.295 ± 0.005 ^c^	1.322 ± 0.201 ^b^	0.382 ± 0.009 ^b^
*Cyanobium gracile* LEGE12431	0.458 ±0.120 ^a,b^	0.921 ± 0.007 ^d^	*i*	*i*
*Nodosilinea (Leptolyngbya) antarctica* LEGE13457	0.730 ± 0.153 ^b^	0.095 ± 0.002 ^a^	*i*	0.319 ± 0.009 ^a^
*Cuspidothrix issatschenkoi* LEGE03282	0.177 ± 0.039 ^a^	0.198 ± 0.008 ^b^	0.728 ± 0.065 ^a^	0.286 ± 0.012 ^a^
*Leptolyngbya-like* sp. LEGE13412	*i*	0.269 ± 0.024 ^c^	*i*	*i*

^1^ Different superscript letters in each column denote statistical differences at *p* < 0.05 (ANOVA, Tukey HSD and unpaired *t*-test performed to compare the IC_50_ values of EtOH extracts); ^2^ Mean ± SD of at least two independent assays; *^i^* radical scavenging did not reach IC_25_ nor IC_50_.

## Supplementary Materials

The following are available online at https://www.mdpi.com/2076-2607/8/2/302/s1. Figure S1: Nitric oxide (NO) production by RAW 264.7 cells in the presence of cyanobacteria ethanol extracts, without stimulation with lipopolysascharide (LPS). Figure S2: Nitric oxide (NO) production by RAW 264.7 cells in the presence of cyanobacteria acetone extracts, without stimulation with lipopolysascharide (LPS). Figure S3: RAW 264.7 cells viability after 24 h of incubation with cyanobacteria ethanol and acetone extracts. Figure S4: Keratinocytes (HaCaT) viability after 24 h and 48 h of incubation with cyanobacteria ethanol extracts. Figure S5: Keratinocytes (HaCaT) viability after 24 h and 48 h of incubation with cyanobacteria acetone extracts.
