# Supplementary material for: Carotenoids from Cyanobacteria: A Biotechnological Approach for the Topical Treatment of Psoriasis
Source: Microorganisms. 2020 Feb 21;8(2):302. doi: 10.3390/microorganisms8020302 (PMC7074968; doi:10.3390/microorganisms8020302)
Supplement: Supplementary file 1 [file microorganisms-08-00302-s001.pdf]

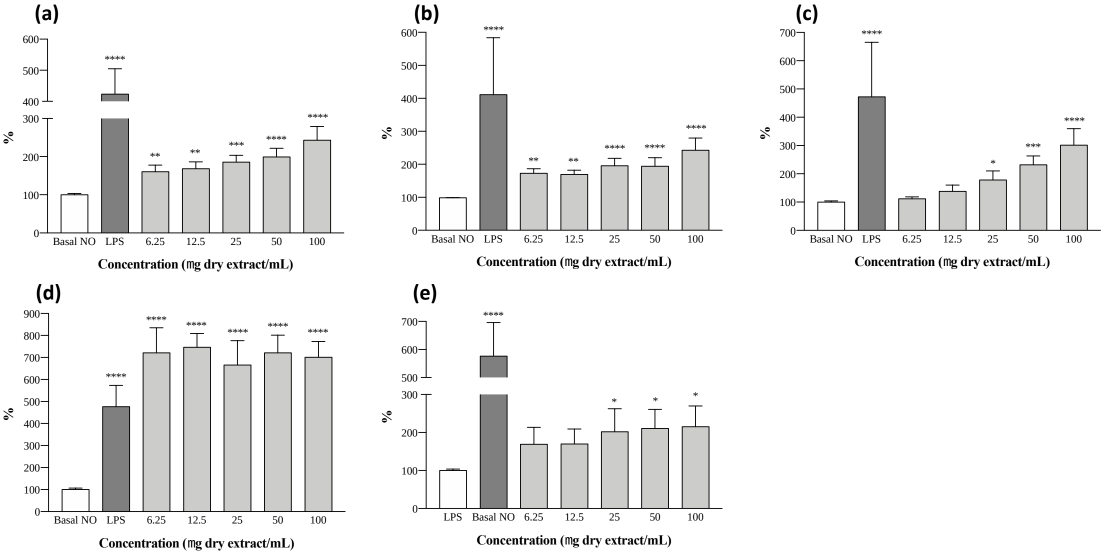

Figure S1. Nitric oxide (NO) production by RAW 264.7 cells in the presence of cyanobacteria ethanol extracts, without stimulation with lipopolysascharide (LPS). **(a)** *Alkalinema aff. pantanalense* LEGE 15481, **(b)** *Nodosilinea (Leptolyngbya) antarctica* LEGE 13457, **(c)** *Leptolyngbya*-like sp. LEGE 13412, **(d)** *Cuspidothrix issatschenkoi* LEGE 03282 and **(e)** *Cyanobium gracile* LEGE 12431. Results are expressed as % of NO relative to control without lipopolysaccharide (LPS) stimulation (Basal NO). LPS represents the NO produced by RAW 264.7 cells with LPS stimulation at 1 μg/mL. Results are expressed as mean ± SD of at least four independent assays, performed in duplicate. *$p<0.05$, **$p<0.01$, ***$p<0.001$ and ****$p<0.0001$ (ANOVA, Tukey HSD).

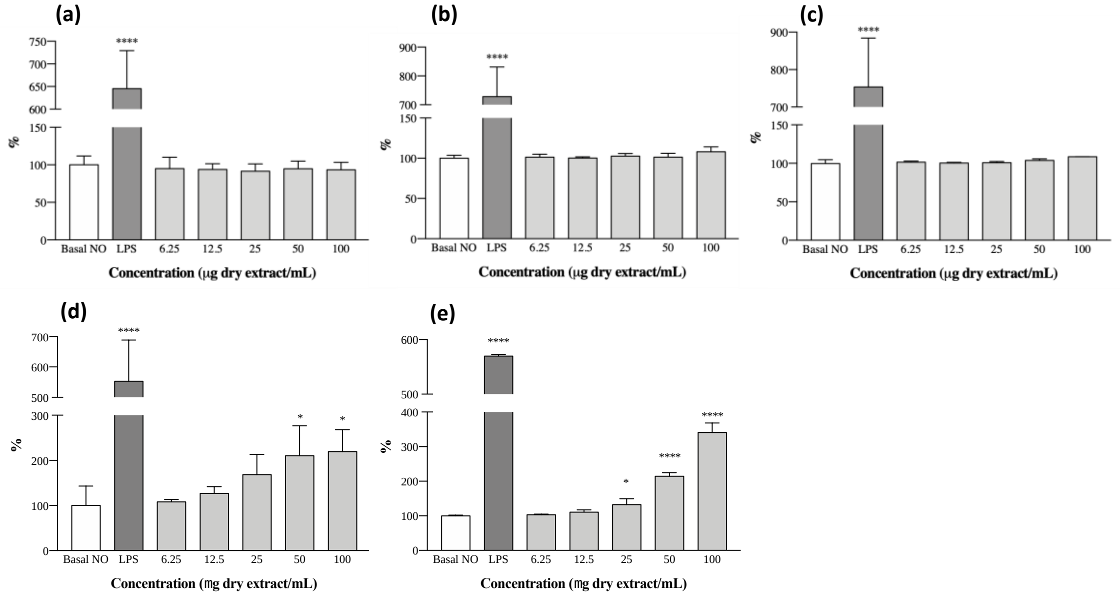

Figure S2. Nitric oxide (NO) production by RAW 264.7 cells in the presence of cyanobacteria acetone extracts, without stimulation with lipopolysascharide (LPS). **(a)** *Alkalinema aff. pantanalense* LEGE 15481, **(b)** *Nodosilinea (Leptolyngbya) antarctica* LEGE 13457, **(c)** *Leptolyngbya*-like sp. LEGE 13412, **(d)** *Cuspidothrix issatschenkoi* LEGE 03282 and **(e)** *Cyanobium gracile* LEGE 12431. Results are expressed as % of NO relative to control without LPS stimulation (Basal NO). LPS represents the NO produced by RAW 264.7 cells with LPS stimulation at 1 µg/mL. Results are expressed as mean ± SD of at least four independent assays, performed in duplicate. **p*<0.05 and *****p*<0.0001 (ANOVA, Tukey HSD).

ETHANOL

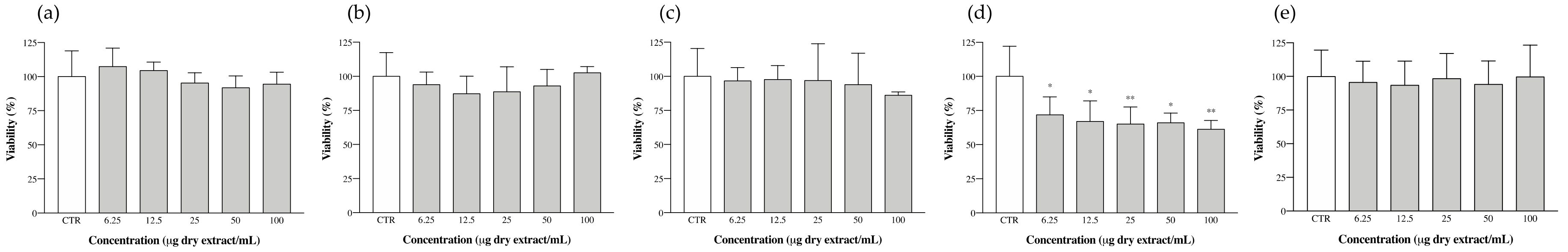

ACETONE

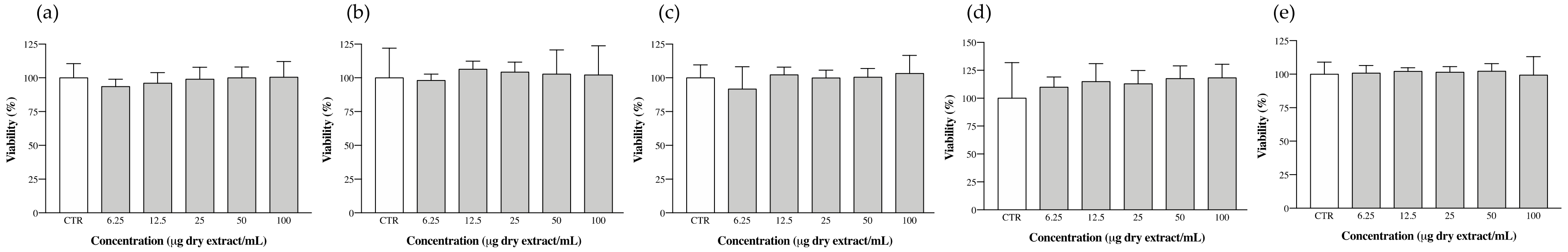

Figure S3. RAW 264.7 cells viability after 24 h of incubation with cyanobacteria ethanol (70% *v/v*) and acetone extracts. **(a)** *Alkalinema aff. pantanalense* LEGE 15481, **(b)** *Nodosilinea (Leptolyngbya) antarctica* LEGE 13457, **(c)** *Leptolyngbya*-like sp. LEGE 13412, **(d)** *Cuspidothrix issatschenkoi* LEGE 03282 and **(e)** *Cyanobium gracile* LEGE 12431. Results are expressed as % of MTT reduction face to the untreated control (CTR). Results are expressed as mean ± SD of at least four independent assays, performed in duplicate. *p<0.05 and **p<0.01 (ANOVA, Tukey HSD).

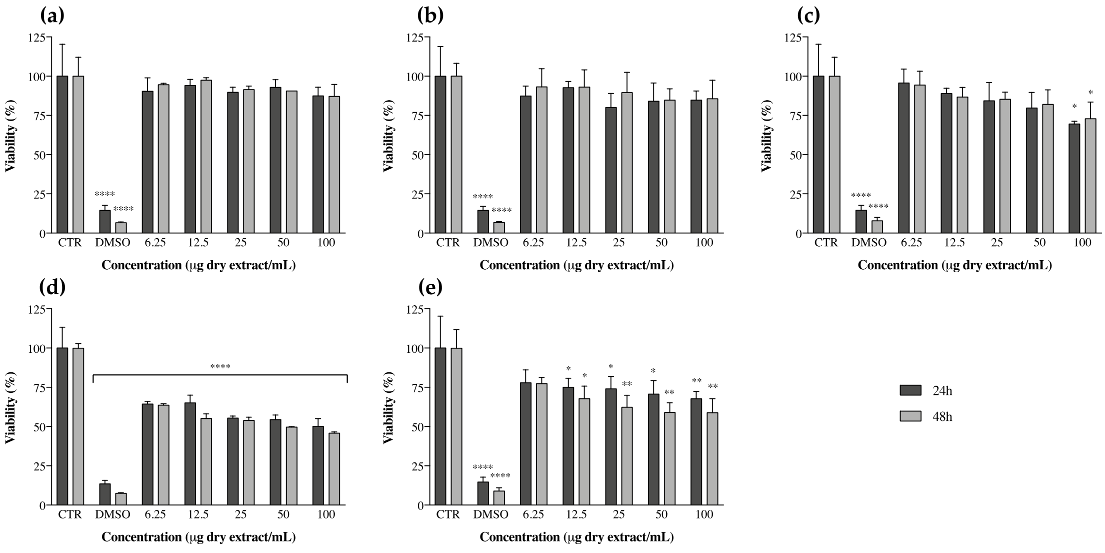

Figure S4. Keratinocytes (HaCAT) viability after 24h and 48h of incubation with cyanobacteria ethanol extracts. **(a)** *Alkalinema aff. pantanalense* LEGE 15481, **(b)** *Nodosilinea (Leptolyngbya) antarctica* LEGE 13457, **(c)** *Leptolyngbya*-like sp. LEGE 13412, **(d)** *Cuspidothrix issatschenkoi* LEGE 03282 and **(e)** *Cyanobium gracile* LEGE 12431. Results are expressed as % of MTT reduction face to the untreated control (CTR). DMSO (20%) represents the positive control. Results are expressed as mean ± SD of at least four independent assays, performed in duplicate. **p*<0.05, ***p*<0.01 and *****p*<0.0001 (ANOVA, Tukey HS)

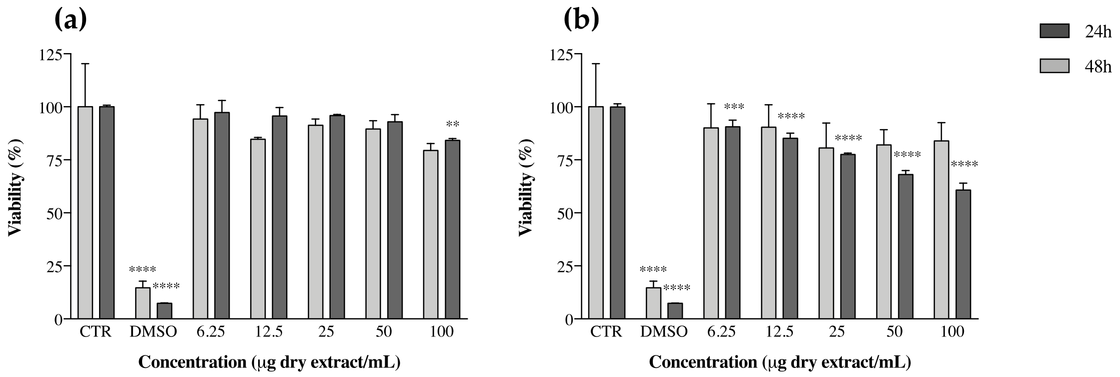

Figure S5. Keratinocytes (HaCAT) viability after 24h and 48h of incubation with cyanobacteria acetone extracts. **(a)** *Cuspidothrix issatschenkoi* LEGE 03282 and **(b)** *Cyanobium gracile* LEGE 12431. Results are expressed as % of MTT reduction face to the untreated control (CTR). DMSO (20%) represents the positive control. Results are expressed as mean ± SD of at least four independent assays, performed in duplicate. ***p*<0.01, ****p*<0.001 and *****p*<0.0001 (ANOVA, Tukey HS)
